# Risk factors for malaria in high incidence areas of Viet Nam: a case–control study

**DOI:** 10.1186/s12936-021-03908-7

**Published:** 2021-09-17

**Authors:** Richard J. Maude, Thang Duc Ngo, Duong Thanh Tran, Binh Thi Huong Nguyen, Dung Viet Dang, Long Khanh Tran, Michael Gregory, Rapeephan R. Maude, Ipsita Sinha, Kulchada Pongsoipetch, Nicholas J. Martin

**Affiliations:** 1 Mahidol-Oxford Tropical Medicine Research Unit, Faculty of Tropical Medicine, Mahidol University, Bangkok, 10400 Thailand; 2 Centre for Tropical Medicine and Global Health, University of Oxford, Oxford, OX3 7FZ UK; 3 Harvard TH Chan School of Public Health, Harvard University, Boston, USA; 4 The Open University, Milton Keynes, UK; 5 National Institute of Malariology, Parasitology and Entomology (NIMPE), Hanoi, Vietnam; 6 Vysnova Partners, Inc (Vysnova), Landover, MD USA; 7 Naval Medical Research Unit-2, Singapore, Singapore

## Abstract

**Background:**

A key step to advancing the goal of malaria elimination in Viet Nam by 2030 is focusing limited resources for treatment and prevention to groups most at risk for malaria transmission.

**Methods:**

To better understand risk factors for malaria transmission in central Viet Nam, a survey of 1000 malaria positive cases and 1000 malaria negative controls was conducted. Cases and controls were matched for age and gender and self-presented at commune health stations (CHS) in Binh Phuoc, Dak Nong and Dak Lak Provinces. Diagnoses were confirmed with microscopy, rapid diagnostic test and PCR. Participants were interviewed about 50 potential risk factors for malaria, which included information about occupation, forest visitation, travel, healthcare-seeking behaviour and prior use of anti-malaria interventions. Participants were enrolled by trained government health workers and the samples were analysed in Vietnamese government laboratories. Data were analysed by univariable, block-wise and multivariable logistic regression.

**Results:**

Among cases, 61.8% had *Plasmodium falciparum*, 35.2% *Plasmodium vivax* and 3% mixed species infections. Median (IQR) age was 27 (21–36) years and 91.2% were male. Twenty-five risk factors were associated with being a case and eleven with being a control. Multivariable analysis found that malaria cases correlated with forest workers, recent forest visitation, longer duration of illness, having a recorded fever, number of malaria infections in the past year, having had prior malaria treatment and having previously visited a clinic.

**Conclusions:**

This study demonstrates the benefits of increased statistical power from matched controls in malaria surveillance studies, which allows identification of additional independent risk factors. It also illustrates an example of research partnership between academia and government to collect high quality data relevant to planning malaria elimination activities. Modifiable risk factors and implications of the findings for malaria elimination strategy are presented.

**Supplementary Information:**

The online version contains supplementary material available at 10.1186/s12936-021-03908-7.

## Background

The World Health Organization (WHO) has set a goal to eliminate malaria in the Greater Mekong Subregion (GMS) by 2030, an objective that Viet Nam agreed to in 2011 [1]. Since 2000 there has been a marked reduction in malaria cases in Viet Nam [2], with an almost 50% reported decrease in indigenous cases from 9331 in 2015 to 4813 in 2018 [3]. Over the same period, malaria transmission has become increasingly focal in Viet Nam [4], with pockets of high incidence within areas of ongoing transmission, particularly in Binh Phuoc province, and parts of the central highlands, which could be sources for the spread of malaria to other regions. It appears from a review of the most recent publicly available data that progress towards malaria elimination has stalled.

From April to September in 2019 there was a 18% increase in confirmed cases nationwide and a 39% increase in *Plasmodium falciparum* compared to the same period in 2018 [5]. As the causes of this increase are not clear, with possible contributors including changes in climate or forest activities (personal communication from National Institute of Malariology, Parasitemia and Entomology (NIMPE)), a detailed study of which groups are now most at risk for malaria transmission is critically needed.

A major challenge for malaria elimination programmes (MEPs) in the developing world is access to funds for national programmes. For many countries the sources of funds for MEPs are external, primarily from the Global Fund to Fight AIDS, Tuberculosis and Malaria [2]. With the decreasing external and domestic funding for malaria programmes in recent years [3] developing countries must necessarily learn to optimize the use of limited resources. Optimizing the use of these resources will require, among other things, targeting of interventions and health services to those groups most at risk of infection, localities of active transmission, and those where malaria is at-risk of being reintroduced.

Gaps remain in the understanding of the epidemiology of malaria in Viet Nam, particularly in the sparse information about groups most at risk, risk behaviours and patterns of population movement which may associate with malaria transmission. These knowledge-gaps hamper efforts to effectively and comprehensively target resources at high-risk populations to accelerate elimination.

To provide evidence-based information for planning of the national malaria strategy, a prospective case–control study was conducted aiming to identify risk factors for malaria and patterns of population movement associated with malaria risk in the highest incidence area of Viet Nam.

## Methods

A prospective, observational case–control study in health facilities in the two highest incidence provinces, Gia Lai and Binh Phuoc, and neighboring Dak Nong province, in central Viet Nam was done from March 2018 to September 2019. Gia Lai and Binh Phuoc provinces have accounted for around half of the national total reported cases in recent years (personal communication from NIMPE). Eligible subjects were patients of at least 6 months of age, living or working in these provinces, and self-presenting to health services in which the treating clinician prescribed a test for malaria. Study sites were the commune health stations (CHS) in the three provinces. CHS are the main source of care for malaria in the public sector [6]. All 117 CHS in the 10 malaria endemic districts of the 3 provinces were included as study sites (Additional file 1: Table S1). Of these, 61 CHS were able to enroll both malaria cases and controls. The planned sample size was 1000 patients with confirmed malaria of any species (“cases”), plus an equal number of malaria negative individuals matched for age and gender. All subjects were tested at the health facility for malaria, as per routine practice, by rapid diagnostic test (SD BIOLINE Malaria Ag Pf/Pv) and/or peripheral blood microscopy (Giemsa-stained slides).

Blood was taken from all patients by finger prick or venipuncture for real-time PCR for *Plasmodium*. Blood samples were sent to the NIMPE laboratories in Hanoi, Viet Nam where real-time PCR was performed to detect parasites and determine *Plasmodium* species. Briefly, *Plasmodium* genomic DNA was purified from dried blood spots (DBS) using QIAamp DNA 96 Blood kit (Qiagen, Cat No./ID: 51161).

Real-time PCR was performed on the Applied Biosystems 7500 Fast Real-Time PCR system (Foster City, CA, USA) and targeted the 18S rRNA gene of *Plasmodium* spp. following a previously published method [7] with a self-designed primer set for detection of *P. falciparum* and *Plasmodium vivax*. The primers and probe used are listed in Additional file 1: Table S2. The reaction mixture (25 µL) included master mix (10 µL) (Quantinova probe PCR kit), forward primer (25 µM; 0. µL), reverse primer (25 µM; 0.35 µL), probe (10 µM; 0.75 µL), purified DNA (5 µL) and nuclease-free water (2.1 µL). The PCR program included 3 min of denaturation at 95 °C followed by 45 cycles of 05-s denaturation at 95 °C, 30-s annealing at 58 °C and 30-s elongation at 72 °C. All results with threshold cycle values (Ct) ≤ 40 were considered positive. The limits of detection of the assay were: 0.5 parasite/µL for *Plasmodium*; 0.5 parasite/µL for *P. falciparum* and 0.8 parasite/µL for *P. vivax* [8].

All enrolled subjects provided written, informed consent. Patients who presented with fever a second-time after an interval of ≥ 2 months were enrolled again.

Subjects were interviewed by trained staff at the CHS about possible risk factors including demographics, place of residence, employment, travel patterns, housing type, use of protective measures against malaria, previous malaria episodes and treatment. Their malaria test result was recorded and, where relevant, a microscope slide saved for later re-examination and parasite quantification. All malaria positive patients were treated in accordance with the Ministry of Health guidelines.

Population data was obtained from the United Nations World Population Prospects 2019 (https://population.un.org/wpp/Download/Standard/Population). Administrative unit shapefiles were Global Administrative Unit Layers downloaded from GeoNetwork (http://www.fao.org/geonetwork/srv/en/main.home).

### Data analysis

Study questionnaire data were compared between cases and controls to identify risk factors for malaria. Demographic data from both groups was compared to data from the most recent population census in Viet Nam to identify age and gender risk groups. Potential risk factors between cases and controls was assessed using multiple logistic regression with the use of dummy variables for categorical data. Block-wise forward selection by thematic group was used to choose predictor variables. Contingency tables were analysed by the Chi Squared method. Time in the forest was assessed by pairwise Wilcoxon test. Median age was compared between two groups using Mann–Whitney test and multiple groups using one-way ANOVA with the Kruskal Wallis test and Dunn’s correction for multiple comparisons. Due to space limitations, the analysis of travel data was confined to identifying risk factors for malaria. More detailed results by demographic and occupation group and extended analyses of the travel data will be published separately.

### Ethics, consent and permissions

This study was approved by the Viet Nam Ministry of Health Ethics Committee and reviewed by the U.S. Department of the Navy Human Research Protection Program in compliance with all applicable federal regulations governing the protection of human subjects (HRPO.NMRCA.2018.0004 and HRPO.NMRCA.2018.0010). All participants provided written, informed consent to participate.

## Results

2000 age, gender and study site-matched cases and controls were enrolled at CHS from February 2018 to March 2019.

### Demographics

**Fig. 1:**
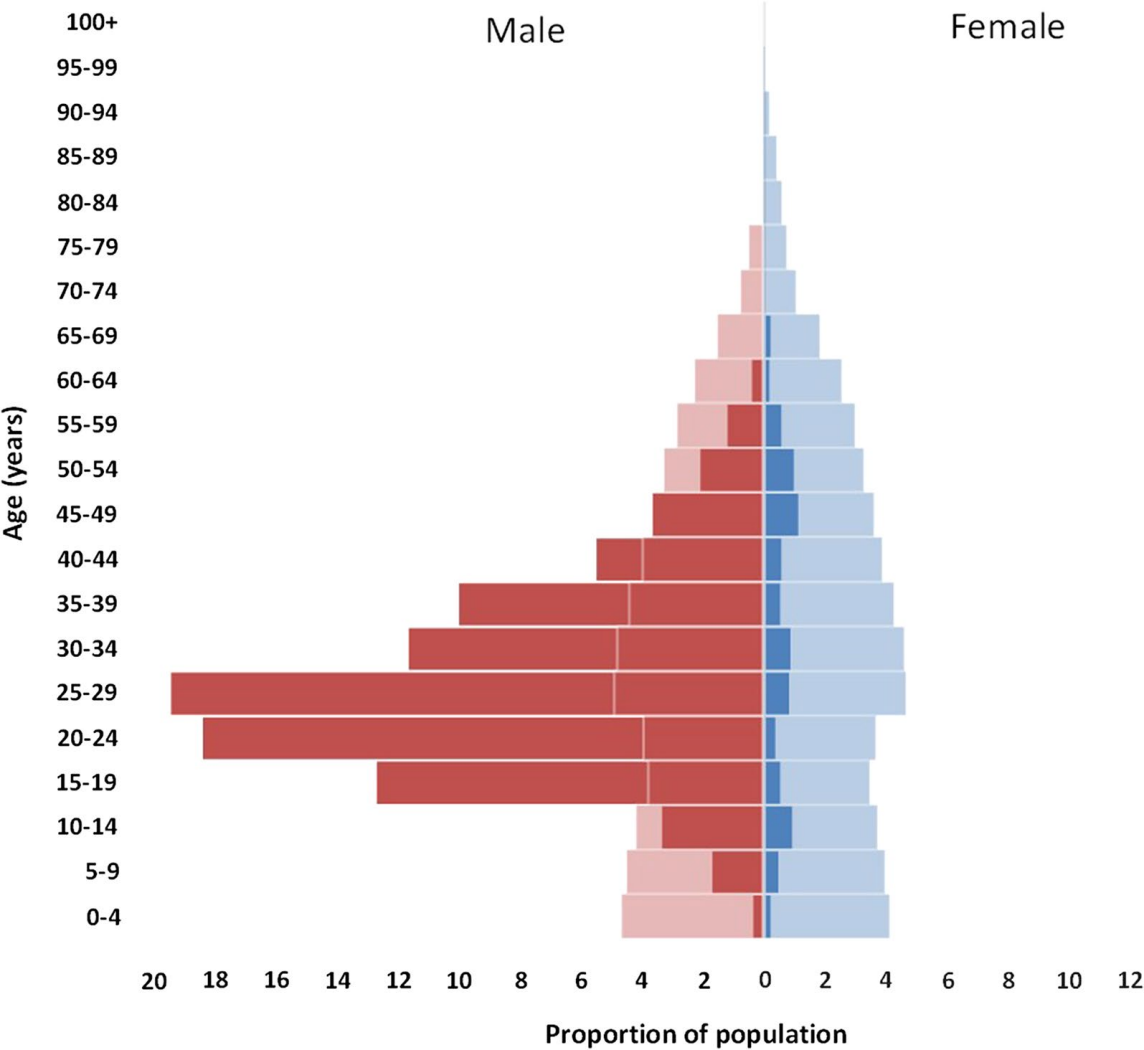
Age and sex distribution of enrolled subjects (dark colours) compared to the general population (pale colours)

The median (interquartile range) of enrolled subjects was 27 (21–36) years and 91.2% were male (Fig. 1). The age-sex ratio of subjects differed from the general population in that there was a higher proportion of males aged 15 to 44 years in the study population.

### Spatial distribution

**Fig. 2:**
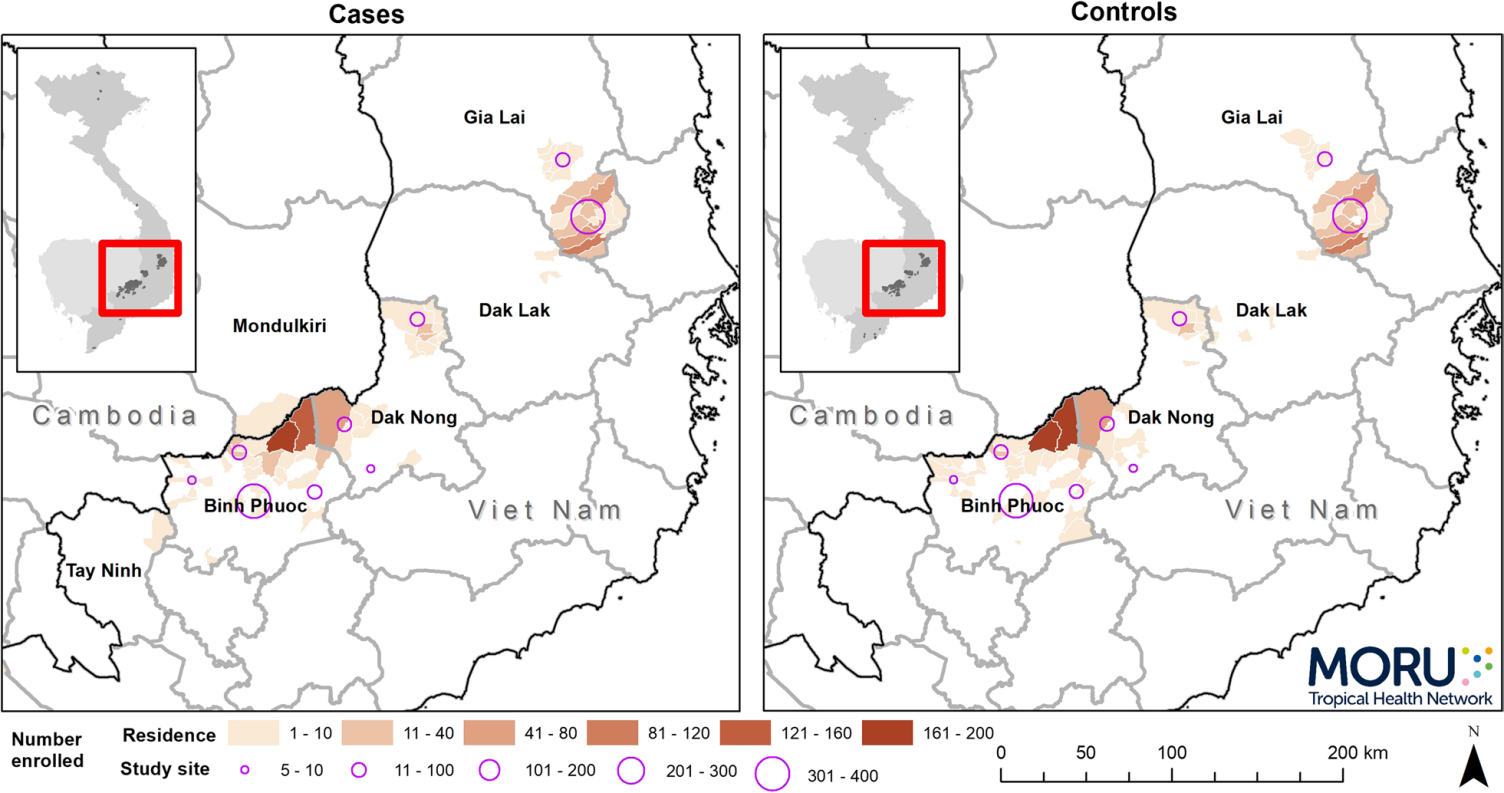
Numbers of cases and controls enrolled by commune of residence and study districts

The spatial distributions of enrolled subjects by place of residence and study site are shown in Fig. 2. Study sites with enrolled subjects were concentrated in locations with higher malaria incidence in the study provinces. Places of residence were grouped in 3 main clusters. The distribution of place of residence among cases and controls was similar. Three cases and no controls said they lived in Cambodia, 1 in Mondulkiri Province and the remainder lived in Viet Nam.

### Parasite species

All identified cases were positive for malaria by microscopy of which 61.8% had *P. falciparum*, 35.2% *P. vivax* and 3% had mixed species infection. All the controls were identified as negative for malaria parasites by microscopy. 1544 (77.2%) subjects also had a rapid diagnostic test performed. Microscopy and RDT gave the same result in 1527 people (98.9%). Eight (0.5%) samples showed discordant results for mixed species *versus* mono-infections, and for eight samples microscopy was negative whereas the RDT result was positive. Real-time PCR was concordant with microscopy in 1893 (94.7%) samples, with discordant results as follows: 31 (1.5%) samples were positive by PCR, but negative by microscopy, in 27 (1.4%) samples different species were identified, and disagreement between mixed vs monoinfection was seen in 48 (2.4%) samples (Table 1).

**Fig. 3:**
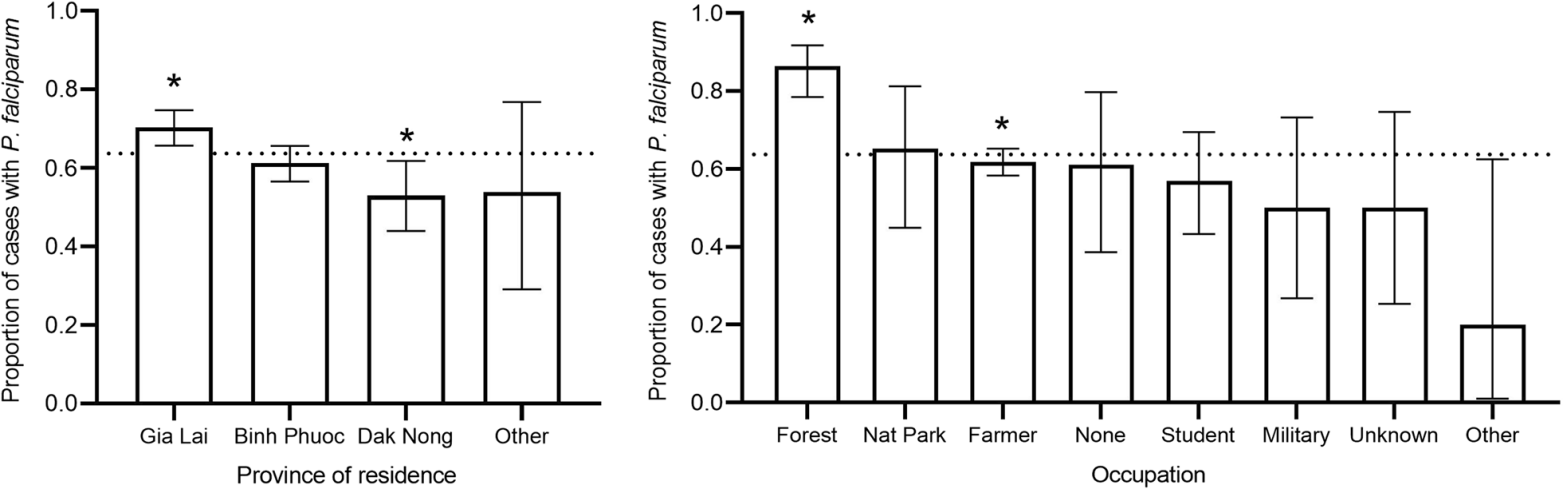
Proportion of cases with *P. falciparum* by **a**) province of residence and **b**) occupation, p < 0.05 for subgroup compared to non-subgroup indicated by *. The dotted line indicates the overall proportion with *P. falciparum*

There were no differences in age or gender distribution between those with falciparum *versus* those with vivax infections. The number of cases of *P. falciparum* infections was higher than *P. vivax* in all provinces (Fig. 3), with Gia Lai having the highest burden of *P. falciparum* (70.4%, p = 0.0016), and lowest in Dak Nong (53.0%, p = 0.0092). There was a difference in species distribution among the reported occupations, with forest workers having the highest proportion of illness due to *P. falciparum* (86.4%, p < 0.0001) and farmers having a lower proportion of falciparum than other occupations (61.8%, p = 0.0269).

### Risk factors for malaria

Of the 50 risk factors assessed, 36 were significant by univariable logistic regression (Additional file 1: Table S3). Twenty-five were associated with being a case, whereas eleven correlated with being a control.

#### Occupation

Of enrolled participants, 1968 (98.4%) stated an occupation (Table 2), the remaining 1.6% reporting staying at home mostly because they were a child (1.4%) or as housewives. Over 80% of all subjects were farmers. The odds ratio for being a case was significantly greater than 1 only for forest workers (OR = 3.129, p = 0.022) and less than 1 only for other/miscellaneous occupations (OR = 0.1258, p = 0.0006).

**Table 1:** Parasite species by microscopy, RDT and PCR

	Pf	Microscopy		Negative	Total
		Pv	PfPv		
PCR					
Pf	**586**	14 17		22	639
Pv	13	**331**	6	9	359
PfPv	19	6	**7**	0	32
PfPm	0	1	0	0	1
Negative	0	0	0	**969**	969
Total	618	352	30	1000	2000
RDT					
Pf	**464**	0	5	7	476
Pv	0	**297**	0	1	298
PfPv	2	1	**23**	0	26
Negative	0	0	1	**743**	744
Not done	152	54	1	249	456
Total	618	352	30	1000	2000

Bold indicates agreement between microscopy and PCR and microscopy and RDT

Pf:  *P. falciparum*, Pv: *P. vivax*, Pm: *P. malariae*

**Table 2:** Occupations and odds ratio for being a case from multiple logistic regression

Variable	Total	Cases	Controls	Odds ratio	p
Farmer	1604 (80.2%)	770 (38.5%)	834 (41.7%)	0.6196 (0.2414 to 1.508)	0.3
Forest worker	156 (7.8%)	127 (6.4%)	29 (1.5%)	3.129 (1.139 to 8.249)	**0.022**
Student	127 (6.4%)	52 (2.6%)	75 (3.8%)	0.4653 (0.1712 to 1.205)	0.12
Other	43 (2.2%)	7 (0.4%)	36 (1.8%)	0.1258 (0.03628 to 0.3966)	**0.0006**
Not working	34 (1.7%)	18 (0.9%)	16 (0.8%)	0.755 (0.2400 to 2.301)	0.62
Military	18 (0.9%)	14 (0.7%)	4 (0.2%)	2.349 (0.5843 to 10.69)	0.24

The area under the ROC curve for occupation was 0.5781 (0.5532–0.6029), p < 0.0001.

1929 subjects (963 cases and 966 controls) gave a location for their work. 168 (16.8%) cases and 99 (9.9%) controls worked in a different commune from where they lived (p < 0.0001); 65 (6.5%) and 36 (3.6%) worked in a different district from where they lived (p = 0.0025); and 55 (5.5%) cases and 32 (3.2%) controls said they worked in a different province from where they lived (p = 0.011).

#### Travel

**Fig. 4:**
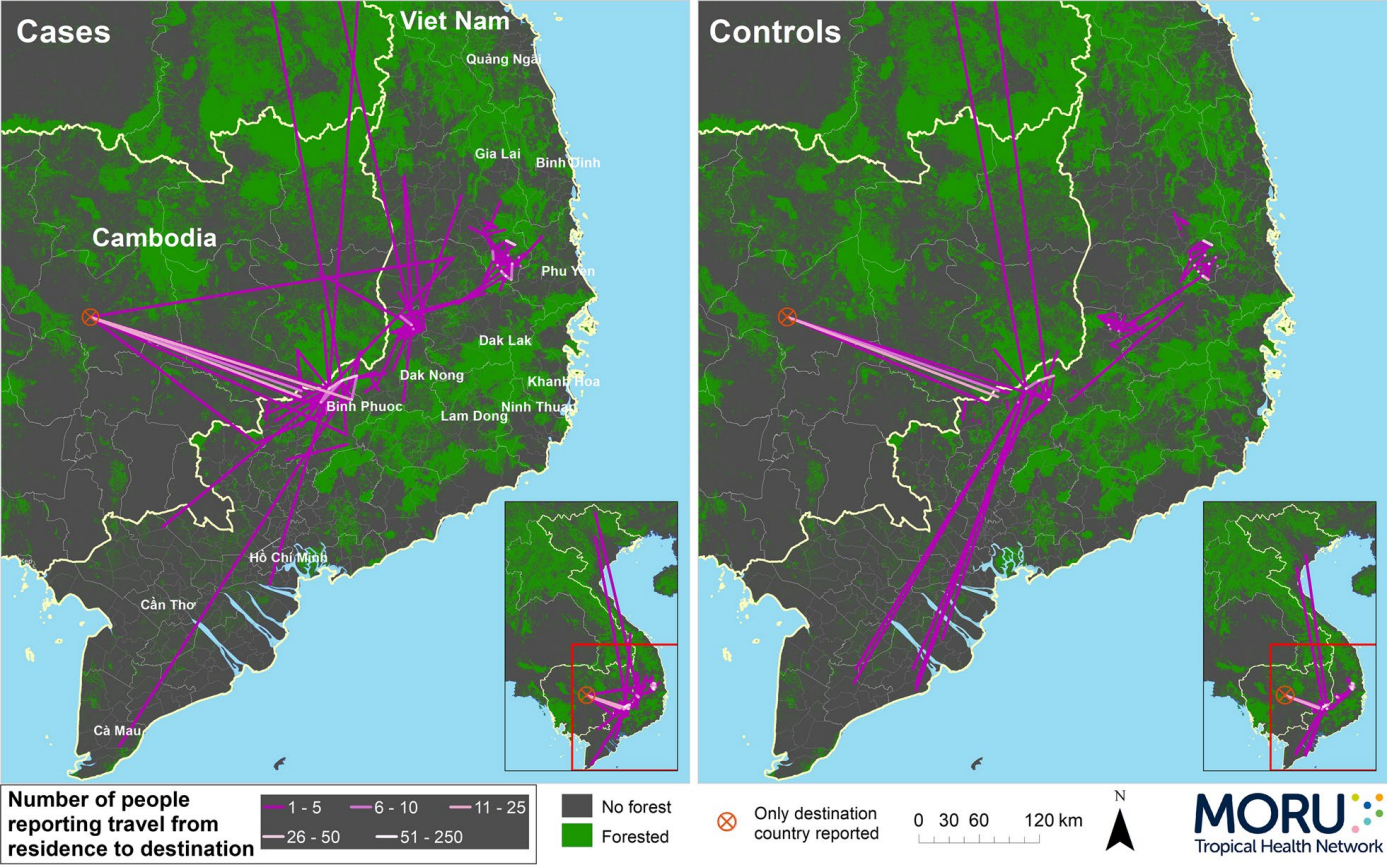
Overall travel patterns of cases and controls at commune level during 2 months prior to enrollment shown as numbers of people travelling between origin and destination

Overall travel patterns are shown in Fig. 4 at commune level. With the exception of Cambodia, most of the travel was within southern Vietnam and mostly around the areas where people were resident. In the 2 months prior to enrolment, 36.8% of cases and 21.1% of controls (p =  < 0.0001) had travelled outside of their commune of residence. 33.0% of cases and 17.8% of controls said they had travelled outside of their commune for purposes other than work (p < 0.0001) and 21.7% and 13.1% (p < 0.0001) for work. The median (IQR) number of nights spent away from home for cases was 6 (0–15) and for controls 0 (0–4), p < 0.0001.

#### Domestic travel

Within Viet Nam, in the 2 months before enrolment, 29.0% of cases and 17.1% of controls said they had travelled to a different commune (p < 0.0001), 11.5% and 5.4% to a different district (p < 0.0001) and 7.8% and 4.4% to a different province (p = 0.0014). For travel outside of their area of residence, cases travelled to a wider variety of areas than controls (39 vs 26 different communes, Fig. 4) in this 2-month period.

**Fig. 5:**
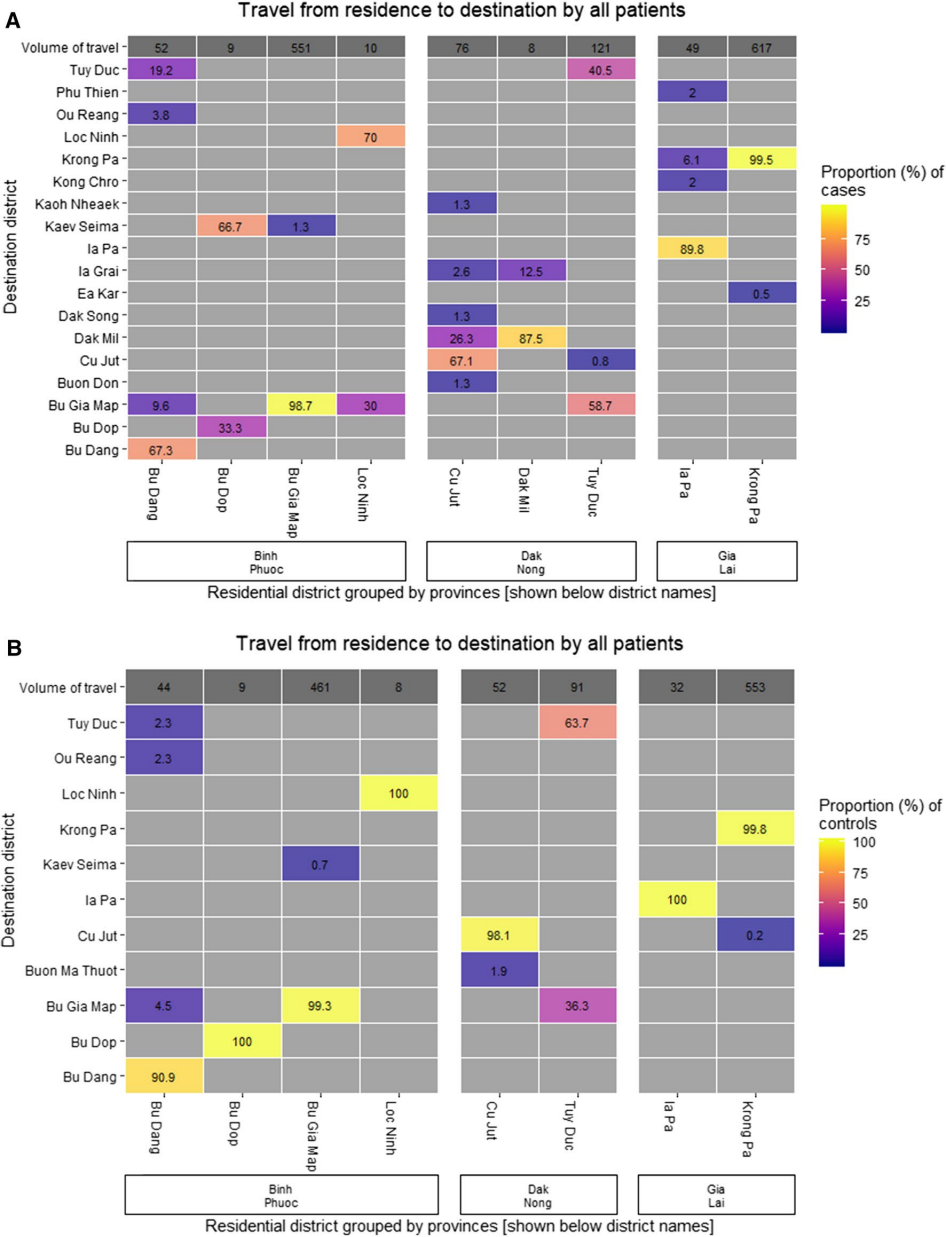
Travel origins and destinations by district for cases and controls. Percentages are of the total number of people resident in that district who reported travel, shown at the top of each column as volume of travel. Only districts with minimum 5 people resident in them are shown. Destination districts are ordered from north to south (top to bottom) and districts of residence are grouped by province and ordered from south to north (left to right)

The top three destination districts for cases travelling outside of their district of residence were Bu Gia Map (n = 58) in Binh Phuoc, and Dak Mil (20) and Tuy Duk (11) in Dak Nong and for controls Bu Gia Map (32), Cu Jut (4) in Dak Nong and Bu Dang (2) in Binh Phuoc (Fig. 5). People who travelled between provinces, districts and communes were older than those who did not (provinces median (IQR) 30 (22–38) vs 27 (20–36) years, p = 0.032; districts median 30 (24–39) vs 27 (20–36), p = 0.0040; communes 28 (23–35) vs 26 (20–36), p = 0.023 and p = 0.0047 with correction for multiple comparisons).

#### International travel

109 people had visited Cambodia, (70 cases and 39 controls, P = 0.0023) within the past 4 years, of which 98 had visited in the previous 2 months (61 cases and 37 controls, p = 0.0129). Only 16 cases and 4 controls reported where in Cambodia they had visited, with all stating Mondulkiri Province. Of the 109, 56 (80.0%) cases and 34 (87.2%) controls had visited the forest; 82 were for exploitation of timber, minerals or catching animals, 5 farming, 2 fishing and 1 hunting. 105 (96.3%) of those who visited Cambodia stated their occupation to be farmer, 3 forest workers, and 1 soldier. Median (IQR) age of international travelers was 29.5 (26–36) years. The median (IQR) time spent in Cambodia for work was 48 (32–56) nights for cases and 32 (28–40) nights for controls, p = 0.0016. For purposes other than work, this was 17.5 (12–24.25) nights for cases and 21 (14.75–28) nights for controls, p = 0.073. 23 cases and 5 controls reported having visited Cambodia between 2 months and 4 years ago. 17 (60.7%) of these were the same people that reported travel to Cambodia in the past 2 months. There was no other international travel reported. Most people who travelled to Cambodia lived in communes close to the international border (Additional file 1: Fig. S1).

#### Travel to forest

**Fig. 6:**
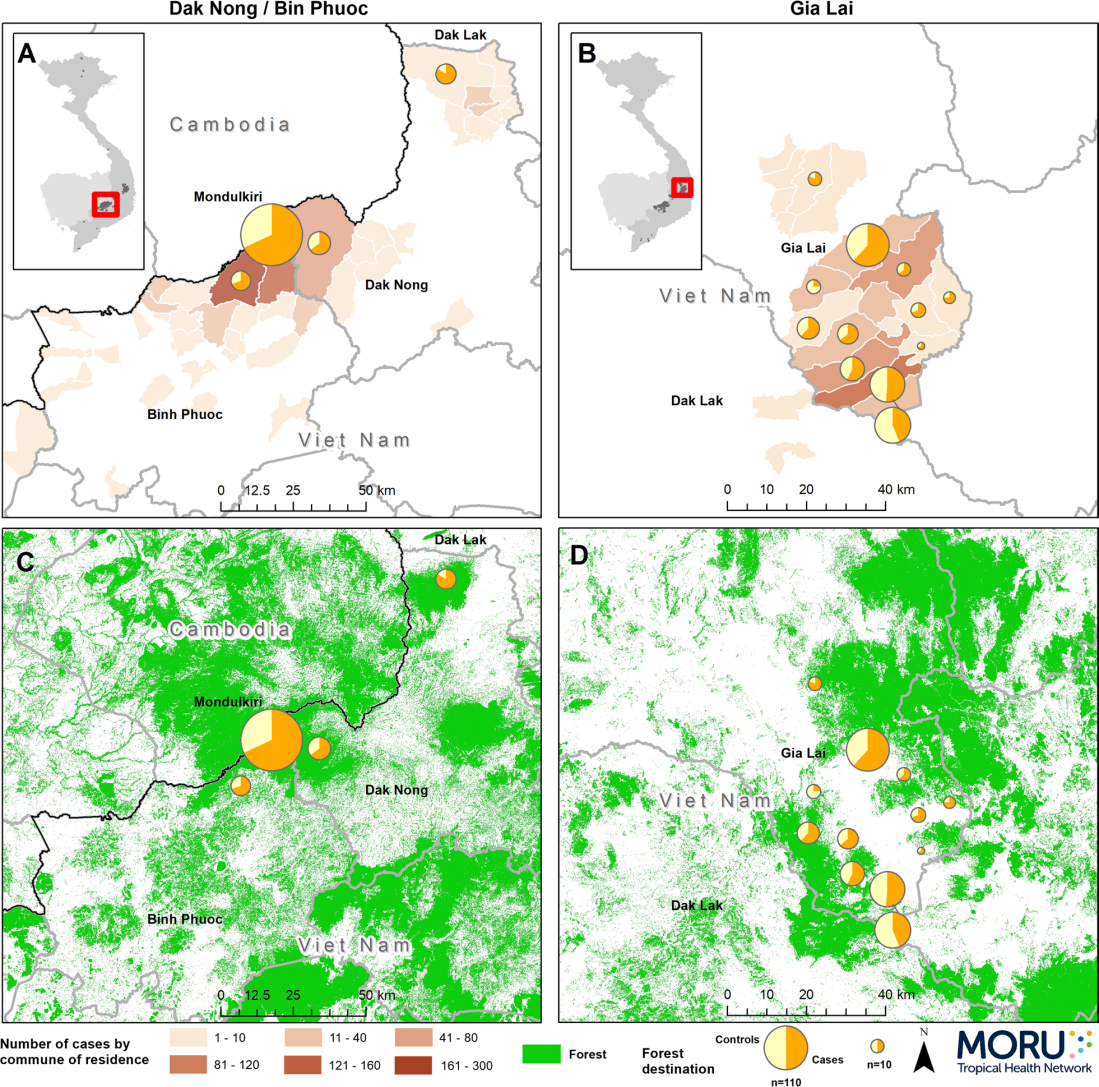
Commune of residence (**A** and **B**) and travel destinations in the forest with forest cover (**C** and **D**) for cases and controls

675 (67.5%) cases and 375 (37.5%) controls had visited the forest in the previous 2 months, p < 0.0001. Of these, 35 (3.5%) cases and 20 (2%) controls said they lived in the forest, p = 0.040. For cases and controls, the places of residence and places visited in the forest are shown in Fig. 6. Of those who said they had visited the forest, 51/640 (8.0%) cases and 31/355 (8.7%) controls had visited Cambodia, 11.7% of cases and 6.5% of controls visited a different province from their place of residence, 38.3% of cases and 35.8% of controls visited a different commune. Of those who said where they had gone in the forest, 12.5% of cases and 7.1% of controls had visited the forest in a different province (p = 0.012), 16.4% and 7.7% a different district (p = 0.0002) and 43.0% and 39.6% a different commune (p = 0.32) from their place of residence. Of the 675 cases who had been to the forest in the preceding 2 months, 62% had *P. falciparum*, 36% *P. vivax* and 2% mixed infection. Of the 325 cases who had not been to the forest, 61.5% had *P. falciparum*, 33.5% *P. vivax* and 4.9% mixed infection.

In Binh Phuoc and Dak Nong provinces, study participants visited the forest in only 2 communes in each province. Three of these communes were adjacent and had the most enrolled cases of all the communes. This area contains Bu Gia Map National Park, an extensive area of natural forests where illegal logging of hardwood trees and hunting and trapping for both subsistence and the illegal wildlife trade are known to take place. In the south of Gia Lai province, people visited forest in more of the communes with 3 predominating. Throughout this area are patches of natural forest and it includes the forested Ea So Nature Reserve along the border with Dak Lak province.

Of the 1050 who visited the forest, 1042 gave a reason for doing so, as summarized in Table 3. There was no association of reason given with whether someone was a case or a control. Area under the ROC curve for reason for visiting the forest was 0.5386 (0.5005–0.5730), p = 0.1350.

**Table 3:** Reasons for visiting the forest and odds ratio for being a case from multiple logistic regression

Variable	Total		Cases		Controls		OR	p
Exploitation*	485	− 46.20%	303	− 44.90%	182	− 48.50%	1.567 (0.7664–3.190)	0.2131
Farming	193	− 18.40%	128	− 19.00%	65	− 17.30%	1.853 (0.8741–3.920)	0.1046
Patrol	133	− 12.70%	91	− 13.50%	42	− 11.20%	2.039 (0.9355–4.444)	0.0713
Fishing	86	− 8.20%	58	− 8.60%	28	− 7.50%	1.95 (0.8581–4.446)	0.1098
Foraging**	67	− 6.40%	42	− 6.20%	25	− 6.70%	1.581 (0.6789–3.697)	0.2869
Business	19	− 1.80%	12	− 1.80%	7	− 1.90%	1.613 (0.5151–5.311)	0.4171
Hunting	17	− 1.60%	12	− 1.80%	5	− 1.30%	2.259 (0.6729–8.445)	0.2002
Other	42	− 4.00%	23	− 3.40%	19	− 5.10%	1.882 (0.4903–8.203)	0.3693
Unknown	8	− 0.80%	6	− 0.90%	2	− 0.50%	3.765 (0.4910–77.89)	0.2576
Total	1050		675		375			

^*^Of timber, minerals or animals

^**^Picking wild vegetables, bamboo shoots, wild fruit or mushrooms

#### Time in the forest

**Fig. 7:**
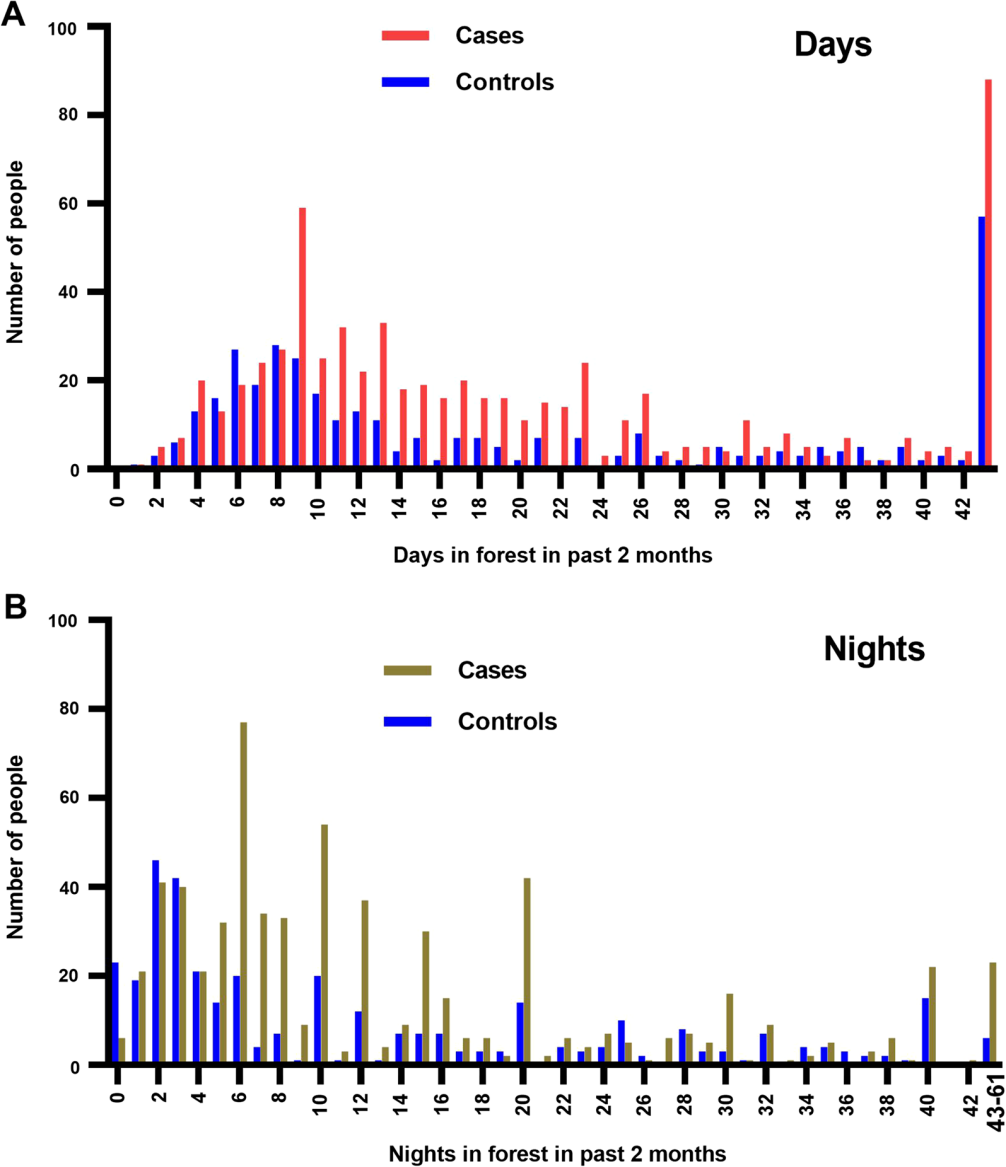
Numbers of days and nights spent in the forest in the past 2 months by cases and controls

Of those who visited the forest in the previous two months, the median (IQR) total number of days on which they were in the forest was 15 (9–27) for cases and 12 (7–32) for controls, p = 0.0064. For days without overnight stays this was 5 (2–9) for cases and 5 (2–12) for controls, p = 0.088 (Fig. 7). For nights spent in the forest, this was 10 (5–20) and 6 (2–20), respectively, p < 0.0001. Multiple logistic regression in a model which included whether someone reported visiting the forest or not, nights spent in the forest and days spent in the forest found the number of nights in the forest to be positively associated with being a case (OR 1.020 (1.008–1.033) p < 0.0009), whereas the number of days was not associated. Area under the ROC curve for this model was 0.6696 (0.6456–0.6936), p < 0.0001 correctly classifying 64.5% of enrolled subjects.

#### Demographics and occupation of forest visitors

The median (IQR) age of people visiting the forest was higher at 28 (22–35) years than those who did not visit the forest at 26 (19–36) years, p = 0.0002. The median (IQR) age of those living in the forest was 29 (20–38) years and this was not different from those who did not live there. A higher proportion of those who visited the forest were male (94.4%) than those who did not visit (87.7%, p < 0.0001), but not different from those who lived in the forest (94.5%). The proportion of people who visited the forest did not vary by month of the study. However, the numbers of days and nights spent per person in the forest were higher in the wet (May–October) than in the dry season (November–April).

#### Travel as a risk factor

In a multiple logistic regression model of all different measures of travel, visiting a different district, visiting the forest and number of nights away were each independently associated with increased odds of being a case (Table 4). Number of nights away was more strongly associated than number of nights in the forest and the two factors were highly correlated, thus, nights in the forest was dropped from the multivariable model. Number of days without overnight stay in the forest was associated with being a control.

**Table 4:** Travel and odds ratio for being a case from multiple logistic regression

Travel variable	Total	Cases	Controls	Odds ratio for case	p value
To different province	122 (6.1%)	78 (7.8%)	44 (4.4%)	0.5238 (0.2582–1.034)	0.0665
To different district	169 (8.5%)	115 (11.5%)	54 (5.4%)	2.212 (1.203–4.232)	**0.0129**
To different commune	461 (23.1%)	290 (29.0%)	171 (17.1%)	0.9255 (0.6048–1.408)	0.7193
International	109 (5.5%)	70 (7.0%)	39 (3.9%)	2.563 (0.9462–8.217)	0.0821
Non-work	508 (25.4%)	330 (33.0%)	178 (17.8%)	0.959 (0.6382–1.446)	0.8407
Work only	348 (17.4%)	217 (21.7%)	131 (13.1%)	3.489 (2.705–4.510)	**< 0.0001**
Number of nights away*	1 (0–9.5)	6 (0–12)	0 (0–3)	1.353 (0.9357–1.957)	0.108
Visit forest	995 (49.8%)	640 (64.0%)	355 (35.5%)	2.759 (0.9833–9.928)	0.078
Live in forest	55 (2.8%)	35 (3.5%)	20 (2.0%)	1.014 (1.001–1.027)	**0.0357**
Days in the forest*	1 (0–5)	2 (0–7)	0 (0–3)	0.9753 (0.9568–0.9940)	**0.01**

^*^Median (IQR)

#### Interventions

**Fig. 8:**
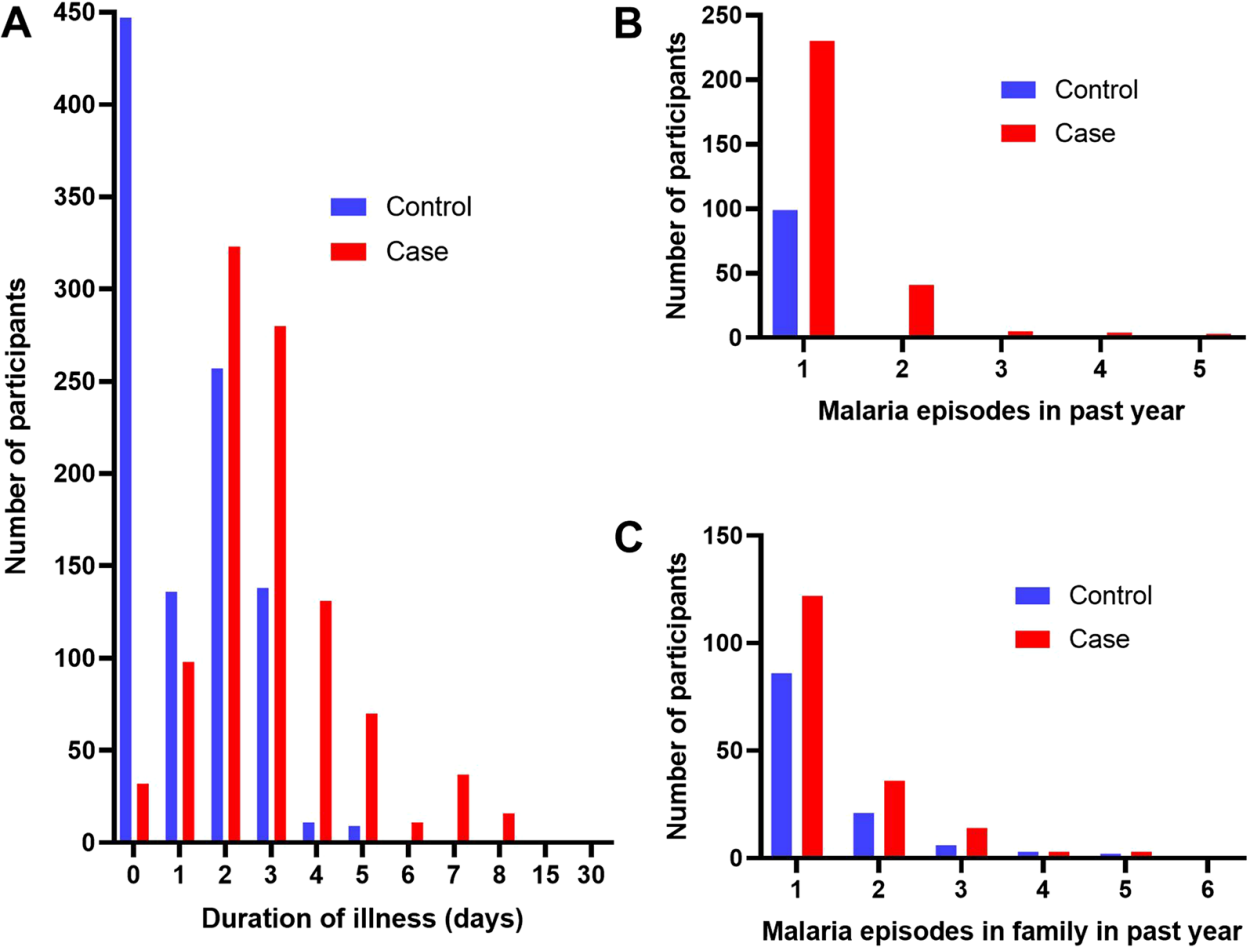
Past malaria infections. **A** Duration of illness, **B** number of malaria episodes in that individual in the past year, **C** number of malaria episodes in the person’s family in the past year

Responses to questions about malaria history, prior treatment and use of healthcare for the present illness, long-lasting insecticidal bed nets, their household and use of personal mosquito repellants are summarized in Table 5. The median duration of illness was longer for cases who also more often had a measured fever and past malaria infections within the previous 1 year compared to controls (Fig. 8). These 3 factors were independently associated with being a case in a model which also included how many times people in their household had had malaria in the past year.

**Table 5:** Use of interventions by cases and controls with results of multiple logistic regression in groups by theme shown with area under the ROC curve for each group

Theme	Variable	Cases	Controls	OR	p value
Malaria history	How long been unwell (days)*	3 (2–4)	1 (0–2)	2.576 (2.346–2.841)	**< 0.0001**
	Had recorded fever	143 (14.3%)	47 (4.7%)	2.172 (1.480–3.231)	**< 0.0001**
	Times had malaria in past 1 year*	0 (0–1)	0 (0–0)	3.779 (2.873–5.031)	**< 0.0001**
	Times people in your house	0 (0–0)	0 (0–0)	0.9848 (0.8096–1.196)	0.8776
	had malaria in past year*				
	Area under ROC			0.8395 (0.8225–0.8565)	**< 0.0001**
Treatment	Had prior treatment	366 (36.6%)	106 (10.6%)	4.869 (3.847–6.206)	**< 0.0001**
	Finished treatment	350 (95.6%)	99 (93.4%)		
	Vomited treatment	36 (9.8%)	19 (17.9%)		
	Area under ROC			0.63 (0.6056–0.6544)	**< 0.0001**
Prior healthcare	Government health center	104 (10.4%)	36 (3.6%)	3.748 (2.536–5.654)	**< 0.0001**
for this illness	Any medical care	368 (36.8%)	212 (21.2%)	0.9816 (0.4626–1.984)	0.9597
	Government hospital	10 (1.0%)	2 (0.2%)	5.744 (1.287–41.41)	**0.0394**
	Pharmacy	310 (31.0%)	184 (18.4%)	2.38 (1.171–5.089)	**0.0197**
	Private clinic	54 (5.4%)	13 (1.3%)	5.66 (2.322–14.76)	**0.0002**
	Area under ROC			0.6283 (0.6039–0.6526)	**< 0.0001**
LLIN	Household has LLIN	908 (90.8%)	911 (91.1%)		
	Per person*	1 (1–1)	1 (1–1)	0.2143 (0.06193–0.6692)	**0.0102**
	Use	729 (80.0%)	911 (92.4%)		
	Age (months)*	7 (6–12)	10 (6–15.5)	0.9748 (0.9628–0.9865)	**< 0.0001**
	Last treated (months)*	6 (6–11)	8 (6–12)	0.9935 (0.9832–1.003)	0.2067
	Has holes	10 (1.1%)	6 (0.7%)	2.321 (0.8358–7.007)	0.1133
	Area under ROC			0.6042 (0.5752–0.6332)	**< 0.0001**
House	Size*	5 (4–6)	5 (4–6)	0.9302 (0.8764–0.9837)	**0.0162**
	Sealed to mosquitoes	8 (0.9%)	7 (0.7%)	1.199 (0.4280–3.437)	0.7274
	Have AC	6 (0.6%)	11 (1.1%)	0.5445 (0.1864–1.442)	0.2344
	Area under ROC			0.5439 (0.5178–0.5701)	**0.0011**
Personal	Use coil	152 (15.2%)	173 (17.3%)	2.392 (1.763–3.265)	**< 0.0001**
protection	Use repellent	198 (19.8%)	382 (38.2%)	0.622 (0.4682–0.8260)	**0.001**
	Use plug-in	181 (18.1%)	425 (42.5%)	0.2842 (0.2138–0.3753)	**< 0.0001**
	Area under ROC			0.6347 (0.6103–0.6591)	**< 0.0001**
	Use LLIN			0.4308 (0.3195–0.576)	**< 0.0001**
	Use coil			2.396 (1.758–3.283)	**< 0.0001**
	Use repellent			0.7364 (0.5514–0.9836)	**0.0381**
	Use plug-in			0.305 (0.2281–0.4051)	**< 0.0001**
	Area under ROC			0.656 (0.6318–0.6803)	**< 0.0001**

Where no odds ratio is shown this indicates that the logistic regression could not be fit for this variable in combination with any others in the group

^*^Median (IQR)

Of the 366 (36.6%) cases who had been treated for malaria in the past year, 347 (94.8%) said they had been treated in government facilities (310 health centres, 37 hospitals and 1 both), 3 in pharmacies, 3 private clinics, 2 by border guards and 11 did not remember. Of those with *P. falciparum* (n = 215 monoinfection plus 3 mixed) 58.9% said they had been given dihydroartemisinin-piperaquine (DHA-PPQ; first-line in national guideline), 16.7% DHA-PPQ plus primaquine, 4.0% chloroquine, 1.8% chloroquine plus primaquine and 24.6% did not know. Of those with *P. vivax* (n = 138 mono-infection plus 13 mixed), 23.2% had chloroquine plus primaquine (first-line in national guideline), 11.9% chloroquine alone, 21.9% DHA-PPQ, 0.7% DHA-PPQ plus primaquine, 0.7% artesunate, 2.7% quinine and 40.4% did not know.

Of the 250 (68.3%) cases who could say the name of the treatment they had been given, 150/247 (60.7%) of those treated in government facilities reported treatment in accordance with the national guidelines. An additional 15.4% (total = 76.1%) were appropriately given DHA-PPQ plus primaquine for falciparum malaria, as per WHO guidance [9]. The remainder said they were given chloroquine alone (10.1%; 7 for falciparum, 16 vivax and 2 mixed), DHA-piperaquine (10.1%; 25 vivax) or quinine (1.6%; 3 vivax, 1 mixed) in health centres and artesunate monotherapy (0.4%; 1 vivax) in a hospital.

One hundred and eleven of the 366 treated cases said they had taken malaria treatment within the preceding 2 months. Most of these had finished treatment despite 9.8% of them vomiting. 304 (83.1%) of the treated cases were tested for malaria prior to starting treatment; all but 4 had a positive malaria test. 310 (84.7%) had been given this treatment in a government health centre, 38 (10.4%) in a government hospital, 3 in a pharmacy and 3 in a private clinic. 106 (10.6%) of the 1000 malaria negative controls also received malaria treatment for their illness. 10 (9.4%) of these were within the past 60 days, and 70 (66.0%) within the past 61 days to 1 year. 96 (90.6%) of these were treated in a government clinic, 5 in a government hospital, 1 in a pharmacy and 1 in a private clinic. 95 (89.6%) of these controls reported having had a test for malaria before treatment and 87 were positive, 4 reported testing negative and 4 did not know the result. Reporting of previous treatment for malaria was associated with being a case; however, the addition of the variables for compliance in completing treatment and/or experiencing vomiting following treatment to this model resulted in a failure to fit the logistic regression.

Of the 111 cases treated in the preceding 2 months, 104 (93.7%) were treated at a government facility, and 82.0% could remember the name of the anti-malarial medication, of which 83.5% matched national (72.5%) or WHO guidelines (11.0%). The remaining 16.5% had been given chloroquine (7.7%; 6 vivax, 1 falciparum), DHA-piperaquine (5.5%; 5 vivax), or quinine (3.3%; 2 vivax, 1 falciparum). One hundred and seven (96.4%) said they had completed the course of medication. Ten controls had malaria treatment within the preceding 2 months; 8 with DHA-piperaquine, 1 chloroquine and 1 could not remember.

Previous healthcare encounters for this illness were reported by 476 (47.6%) cases and 248 (24.8%) controls (p < 0.0001). 150 of these were with government facilities and 720 with the private sector. 4 had been to only 1 type of healthcare facility, 699 had been to 2 different types, 20 to 3 and only 1 to 4 facilities. All those who went to a government facility also said they had been to the private sector. In multiple logistic regression, having previously attended a government health centre, government hospital, pharmacy or private clinic were all independently associated with being a case.

Over ninety percent of households had an LLIN with a median of 1 per person; there was no difference in household possession of LLINs between cases and controls. Controls were more likely to have an older LLIN than cases and less than 1% overall were self-reported to have holes. Only three people (2 cases and 1 control) did not think their bed nets had been treated with insecticide and 29 (18 cases, 11 controls) did not know. The household size (number of people in the house) and whether homes were reported by participants as sealed to mosquitoes or had air conditioning were not different between cases and controls. Use of mosquito repellent cream or lotion and plug-ins was more likely in controls (approximately 40% in controls compared to 20% in cases), but the use of coils was similar in both (15.2% in cases and 17.3% in controls). In a multivariable model of coils, repellants and plug-ins, use of coils was associated with being a case and repellents and plug-ins were associated with being a control. This may be because cases who did not use a coil (84.8%) were less likely than controls to use repellents (9.2% vs 29.9%) or plug-ins (8.0% vs 34.6%). Adding possession of bed nets into the model did not change the direction or significance of the results.

#### Combined risk factors for malaria

**Fig. 9:**
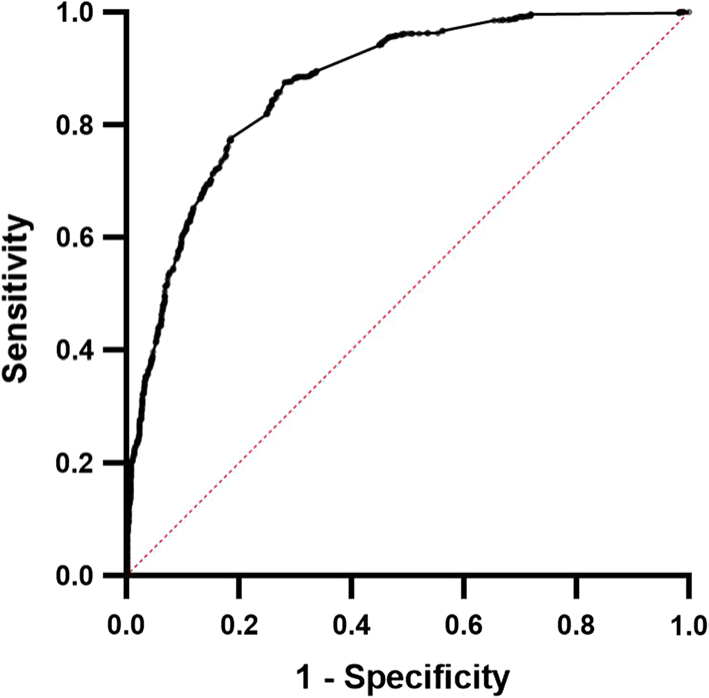
ROC curve for the best fitting multiple logistic regression model shown in Table 6

Upon combining the different groups of significant risk factors together, and adding and removing them one at a time, it was found that ten variables (Table 6) gave the best discrimination between cases and controls with an ROC of 0.8712, p < 0.0001 correctly classifying them in 78.8% (Fig. 9). The negative predictive power of this model was 81.2% and positive predictive power 76.7%. All associations were positive except for occupation *other* (i.e. not forest worker, farmer, student, not working or military) and number of days in the forest. This is in contrast to simple logistic regression, which found more days in the forest (OR 1.030, p < 0.0001) and more nights in the forest (OR 1.042, p < 0.0001) to be associated with being a case. Adding whether someone had been to the forest to the model reversed the direction of association with number of total days away and made the association with number of nights non significant.

**Table 6:** Results of best performing logistic regression model for cases vs controls

Variable	Odds ratio for case	p value
Occupation forest worker	3.533 (2.203–5.836)	**< 0.0001**
Occupation other	0.2247 (0.07564–0.5724)	**0.0034**
Visited the forest	3.23 (2.376–4.404)	**< 0.0001**
Number of days in the forest	0.9762 (0.9661–0.9863)	**< 0.0001**
How long been unwell (days)	2.549 (2.308–2.827)	**< 0.0001**
Had recorded fever	1.766 (1.155–2.735)	**0.0096**
Times had malaria in past 1 year	1.541 (1.103–2.219)	**0.0155**
Had prior malaria treatment	2.976 (1.950–4.530)	**< 0.0001**
Attended government health center	1.952 (1.211–3.203)	**0.0069**
Attended private clinic	2.943 (1.488–6.183)	**0.0028**
Area under ROC curve	0.8712 (0.8559–0.8864)	**< 0.0001**

## Discussion

This large case–control study found 38 risk factors associated with being infected with, or protected from, malaria in Viet Nam. Of these, when combined in a model, 10 independent risk factors, excluding age and gender, were able to correctly classify 78.8% of participants as cases or controls. This study was designed to include matching of age and gender between cases and controls as a strong association of malaria risk with male gender and being a young adult was evident from independent analysis of routine malaria programme data (personal communication from NIMPE) and prior research [10, 11]. Matching of cases and controls in this study was intended to increase the power to detect other risk factors that are age or gender specific. The association of malaria risk with male gender and young adulthood has been shown elsewhere in the GMS in recent studies, particularly those measuring parasite prevalence [12–15]. This correlation is thought to be due to young males spending more time travelling and working in the forest which are focal points of malaria transmission. In some locations in the GMS, clinical episodes of malaria are more common in children [16].

This study investigated a large number of possible risk factors using a block design for the regression analysis to identify which risk factor within each category were most discriminatory. This analytic method has the advantage over the alternative of a stepwise analysis of all individual risk factors in that the range of questions within each theme can first be narrowed, allowing multiple different risk categories to be identified. The number of possible combinations of the 50 variables is also very large with a high chance of interactions among the variables, which would render analysis across all variables infeasible. The limitations of stepwise regression for large datasets has been described in the literature [17]. Instead, a stepwise analysis was conducted within each theme using expert knowledge to decide which variables make sense to combine followed a stepwise analysis of the discriminatory variables from each theme. It is possible that this method may have missed some individual variables which could have improved the final multiple regression model whilst not appearing significant in the analysis of each block. It is likely that any additional contribution of these variables to the model would be very marginal, however.

The final output from the multiple logistic regression cannot be directly related to causality, as the analysis yields purely statistical associations. This is because variables that dropped out of the multivariable model could be more closely related to causality whilst being less strongly associated with the outcome than risk factors which remained in the model. Variables that are positively associated with being a case in a univariate or block-wise analysis can be associated with being a control when combined with additional variables. An example is the number of days spent in the forest, which was slightly higher among cases, but predicted in the opposite direction in the final model. This is because other variables in the model accounted for all of the positive association of this variable with being a case. For this reason, the output of a multivariable model like this, although of academic interest, is of limited value for guiding national program planning as it only works if all variables are included and it does not give information about the relative importance of individual risk factors in isolation. It is, however, of value in identifying from particular sets of variables from different domains which combination are the best predictors of malaria risk and this could be used to help guide what information to collect in future studies.

Block-wise and univariate analyses are more informative to identify individual risk factors and risk groups. In this study, visiting the forest was strongly associated with being a case, with a higher number of nights in the forest increasing that risk. Forest workers were the occupation group most strongly associated with malaria, however, these comprised only 7.8% of all cases. An additional sixty percent of cases visited the forest, but did not work there. Of note, there was no association between the different activities in the forest and risk of being a case.

Approximately a third of cases had not been to the forest in this study during the 2 months prior to diagnosis, during which almost all new infections should have occurred [18, 19]. One possibility is that some of these cases were recurrences of *P. vivax* malaria from hypnozoites from a previous malaria episode. However, there was no difference in the proportion with *P. vivax* between those who had and those who had not been to the forest suggesting this not to be the case. Another more likely possibility is that these cases were infected elsewhere, either in their village of residence, village transmission is a possibility due to the observation of cases in children under 10 years of age (Fig. 1) who are not likely to visit the forests. It is also possible these cases were infected in a location they do not consider to be forest or they failed to recall having actually been to the forest. Previous studies have found a strong association between forest visits and malaria in Viet Nam [10, 20] and have highlighted the challenges of reducing transmission among forest-goers. These challenges include that people visiting the forest are less likely to use a bed net when doing so and that much of the biting occurs before sleeping time [21].

Other major risk factors for malaria from this study included duration of illness, confirmed fever and prior treatment for malaria, as well as having previously attended a government health centre or private sector facility for this illness. These may be an indication of healthcare seeking behaviour; people may be shopping around other healthcare providers before visiting the CHS, hence presenting late in their infection course. A plausible explanation is that their malaria infection is being missed on the initial encounter, or that they are failing treatment due either to inappropriate or insufficient anti-malarials, or they are infected with anti-malarial resistant strains. Of the cases who had received anti-malarial treatment within the preceding two months, almost all were treated at a government health facility and almost all took the full course of medication as prescribed. Over 80% of those who could remember their treatment were managed in accordance with the national or WHO guidelines. This suggests insufficient or inappropriate anti-malarials were not a major problem. This relies, of course, on accurate recall of details of treatments received by individuals but the information was volunteered by participants, without prompting, so is likely to be accurate. Around a quarter of enrolled people had attended a pharmacy for this illness and just over 3% a private sector health facility. However, none reported definitely receiving an anti-malarial from these sources. This may be due to a reported misconception that pharmacies are not authorized to provide anti-malarials, whereas they are able to provide medication for those with a prescription [6].

There are a range of implications of these findings for NIMPE malaria management and elimination. This study confirms that males of working age were at higher risk for malaria than other demographic groups; over 90% of cases were male in this study. Secondly, independent of age and gender, people who spent time in the forest in the two months preceding presentation to a clinic, for any reason, were at higher risk of being a malaria case. The malaria risk was even higher for those who spent more nights away from their home and for those who worked in the forest. It should be noted that over 80% of the subjects who visited the forest did not work in the forest. Of the reasons given for visiting the forest, no factor further increased the risk of malaria above that of forest-goers in general. This implies that NIMPE should target interventions at anyone who visits the forest as opposed to specific occupations. Particular attention should be paid to males of working age, people who travel overnight for longer periods and those who work in the forest.

People who had malaria in the past year were also at a higher risk for infection, as were those with household members who had malaria in the past year and those who had previously received anti-malarial treatment at any time. This suggests there are particular groups of people who are being repeatedly infected through high risk behaviours. The number of previous episodes of malaria was independent of whether they had visited the forest in the previous 2 months. This could be because these people had visited the forest previously or because they were being infected elsewhere. It would be informative to explore in more detail about these multiply infected people to identify if their previous diagnoses were confirmed and whether they cluster geographically which could indicate potential transmission hotspots. It may also be worthwhile, targeting these people and households to educate them about malaria risk and ensure they are using healthcare services and personal protection measures optimally.

The identified risk factors are helpful at a population level to identify which groups to target with more intensive interventions and/or to help guide allocation of limited resources e.g. ensuring access to diagnostic and treatment services, LLIN distribution, personal protection measures or targeting of indoor residual spraying. This is particularly pertinent as numbers of cases decline with consequent reductions in funding for malaria elimination [3]. Knowledge about who is at risk can also help to guide delivery of audience-specific public health messaging about malaria prevention and working with these groups to design and implement context appropriate measures, such as hammock bed nets [22] or mobile outreach teams (MOTs).

Another potential application of these findings is to identify modifiable risk factors for malaria. This study found a range of such risk factors, which could be addressed by specific interventions. These could include reducing forest visits and avoiding or minimizing overnight stays in the forest, particularly in areas where the proportion of cases was highest; for example, Bu Gia Map National Park. This has been attempted elsewhere with varying success e.g. through enforcing logging and timber export bans [23]. Just under half the people in this study who visited the forest cited exploitation of timber, minerals or animals as the reason for doing so. As this study was not able to quantify what proportion was for logging, particularly illegal logging, the potential impact of a strictly enforced logging ban on malaria in Viet Nam cannot be determined. Although use of LLIN was high, this study did not collect information on use of personal protection measures specifically in the forest and further work would be required to explore this in detail.

Other potentially modifiable risk factors identified included longer duration of illness and the presence of recorded fever. This is of concern as longer duration of malaria infection could lead to increased transmission before treatment clears parasites from the blood. Early diagnosis and treatment of malaria (EDTM) has been a key component of national malaria strategy in Viet Nam for many years [24].

Community engagement and education to re-emphasize the importance of EDTM and encourage and support people with a fever to seek medical care as early as possible would be beneficial to address this as well as control for other emerging infectious diseases, such as novel coronavirus.

LLIN coverage and usage in the study population was very high, most LLIN were less than 1 year old and very few had holes. This suggests the LLIN distribution programme is functioning well in Viet Nam which is reassuring as it accounts for the majority of external funding for malaria elimination. Smaller numbers of people used other bite prevention methods and tended to use multiple methods or none at all. Repellents and plug-ins were protective against malaria, but coils were not. Unfortunately, due to space limitations, data on use of LLIN or other measures whilst in the forest was not collected.

This information is of limited use for clinical management of individuals as all possible malaria cases are required to have a confirmatory diagnostic test before treatment. Having a pre-test probability of malaria based on risk factors would not change this for people living in endemic areas as testing should be based on the presence or absence of fever without an alternative diagnosis. However, for locations where malaria transmission is very low, it becomes inefficient to test everyone with a fever for malaria. The WHO recommends testing should be based on whether a person may have been exposed to malaria, using history of, for example, travel to a malaria endemic area without protective measures together with a fever or history of fever with no other obvious cause [25]. A more nuanced approach could be to include some of the more strongly associated risk factors identified in this study to develop a screening questionnaire, either as a checklist to derive a score or to assign an individual level pre-test probability of malaria to guide testing. This could be used to help guide who should undergo a diagnostic test as part of passive case detection or even as part of active case detection strategy to optimize use of limited testing resources. Scoring systems based on symptoms are not recommended by the WHO, as they can be complicated to implement and supervise and the key features may be different in different locations [25]. Similarly, risk factors are likely to be different in different locations. Even if it was possible to implement, such a system may only be of use in areas where testing rates are low as it could increase appropriate testing of higher risk individuals thus improving sensitivity of the surveillance system. In areas where testing rates are already high, there is a risk that it could reduce sensitivity by excluding some individuals from testing with the benefit of increased specificity.

The rate of submicroscopic infection was low in this study, with 31 out of 1031 PCR positive individuals being microscopy negative. This differs from other studies in Viet Nam and other low transmission settings which have found the majority of infections to be submicroscopic and/or asymptomatic in cross-sectional surveys [26]. This is probably because all individuals in this study were symptomatic with likely higher parasite burdens than those who are asymptomatic, and were thus more likely to be detectable by microscopy than the general infected population. Differences in sensitivity of PCR and/or microscopy are less likely explanations as the PCR in both studies was done by NIMPE (although previously done by semi-nested PCR, the sensitivity was the same or lower than nested PCR—personal communication) and microscopy was the routine diagnostic methodology used in Viet Nam.

The methodology used had several strengths worth highlighting. Diagnostic testing to identify cases and controls was robust, being done by microscopy and confirmed by PCR in all cases. Microscopy performed very well against PCR with minimal discrepancy. All interviews were done by healthcare workers with relatively little research experience and training. This was supported by academic partners without needing to employ large numbers of field staff. The study materials were developed and optimized by the study partners prior to roll-out to ensure maximal clarity and acceptability of the data capture form and high quality of the information recorded. The data capture form itself was kept as short as possible to minimize workload whilst still collecting sufficient detail. The results suggest relevant questions were properly selected as the model using data from the shorter data capture form was able to correctly identify nearly 80% of cases and controls. The most resource intensive part of the data management and analysis was of the travel data due to the range of different types of travel and amount of geographic information collected. This was made more efficient by use of a standardized travel survey format, which had already been used in other studies across the GMS. This was then entered into a previously developed data processing pipeline and analysis framework [27]. Taken together, these also confer the advantage that this study is relatively easy and cost efficient to repeat and scale up in future in this or other locations. A final strength is that the study was run in close collaboration with NIMPE who are the main beneficiary of the results. Thus very early discussions could be had about the implications as results were generated.

Limitations of this study include that the quality of the data relied on the participant’s accuracy of recall and willingness to disclose information. This is one reason this study focused on travel within the previous 2 months as recall over longer periods can be notoriously unreliable. It is likely that there are items that people were not willing to discuss, such as illegal motivations for visiting the forest, unofficial employment or travel to sensitive locations or illicit crossing of international borders. In some cases, study participants may have adjusted their responses to fit with what they thought the interviewer (a government health worker) wanted to hear, for example they may not want to disclose about use of health services in the private or informal sector. The study sites were all CHS so the study would have missed people who only attended elsewhere including the private sector and larger hospitals. This is particularly a concern for migrant populations whose access to health services is restricted by the Law on Residence which limits health insurance cover to those with permanent registration status [28]. However, it is thought that the vast majority of people with malaria in Viet Nam access health services via CHSs [6]. The quality of the data also relied on the training of the interviewer. For most variables, the data was of extremely high quality and complete. The level of detail for a few variables was constrained, however, by grouping responses into categories at the time of data collection. One example is the cited reasons for visiting the forest where a breakdown of the terms “exploitation” and “foraging” was not available. A further limitation is that age and gender were identified as risk factors by comparison with census. It may be that people living in the malaria endemic areas have different age and gender profiles from the census. However, it is unlikely that they matched the profile of malaria cases of being almost all male and of working age.

This study collected detailed information on travel and it was only possible to provide a summary here, the focus being on identifying risk factors for malaria. A follow-on paper with a more detailed descriptive and modelling analysis of travel and the impact on malaria distribution in Viet Nam will be published separately.

## Conclusion

This large case–control study identified 27 factors associated with increased risk and 11 with decreased risk of malaria in Viet Nam. The results can be informative at the population level for helping to guide provision and targeting of healthcare resources and malaria prevention interventions at particular groups, and at the individual level to help guide decisions about who to test and to identify remediable risk factors. Potential actions by NIMPE based on the findings are under discussion and have the potential to help accelerate the elimination of malaria in Viet Nam.

## Supplementary Information

**Additional file 1: Table S1.** Study sites. **Table S2.** Primers used in real-time PCR. **Table S3.** Results of simple logistic regression with odds ratio for being a case. **Figure S1.** Commune of residence of cases and controls who visited Cambodia (A) and forest distribution (B).

## Data Availability

The datasets generated and analysed during the current study are not publicly available as they belong to the National Institute of Malariology, Parasitology and Entomology and Mahidol Oxford Research Unit. Raw identifiable spatial data cannot be shared. De-identified non-spatial and aggregated spatial data from this study will be available to researchers whose proposed purpose of use is approved by the data access committee at Mahidol Oxford Tropical Medicine Research Unit and after discussion and agreement from the National Institute of Malariology, Parasitology and Entomology. Inquiries or requests for the data may be sent to datasharing@tropmedres.ac.
