# Supplementary material for: Risk factors for malaria in high incidence areas of Viet Nam: a case–control study
Source: Malar J. 2021 Sep 17;20:373. doi: 10.1186/s12936-021-03908-7 (PMC8446736; doi:10.1186/s12936-021-03908-7)
Supplement: Supplementary file 1 — Additional file 1: Table S1. Study sites. Table S2. Primers used in real-time PCR. Table S3. Results of simple logistic regression with odds ratio for being a case. Figure S1. Commune of residence of cases and controls who visited Cambodia (A) and forest distribution (B). [file 12936_2021_3908_MOESM1_ESM.docx]

## Supplementary Information

## Table S1. Study sites.

| Province | District | Total CHC | CHC which enrolled |
| --- | --- | --- | --- |
| Gia Lai | Krong Pa | 14 | 12 |
| Gia Lai | Ia Pa | 10 | 8 |
| Gia Lai | Krong Chro | 15 | 0 |
| Bình Phươc | Bu Gia Map | 8 | 5 |
| Bình Phươc | Bu Dang | 16 | 9 |
| Bình Phươc | Bu Dop | 8 | 4 |
| Bình Phươc | Loc Ninh | 18 | 12 |
| Dak Nong | Cu Jut | 9 | 5 |
| Dak Nong | Tuy Duc | 6 | 2 |
| Dak Nong | Đắk R' Lấp | 12 | 4 |

Table S2. Primers used in real-time PCR.

| Plasmodium species | Name | Sequence |
| --- | --- | --- |
| All | PAN-R | TGTTGAGTCAAATTAAGCCGCAA |
| All | PAN-F | TTAGATTGCTTCCTTCAGTRCCTTATG |
| All | PAN-Probe | **FAM**-TCAATTCTTTTAACTTTCTCGCTTGCGCGA- **BHQ1** |
| *P. falciparum* | Fal-F | CTCTTCAATATGCTTTTATTGCTTTTGAGA |
| *P. falciparum* | Fal-R | GCTTATTCATATTTGTTATTCCATGCTGTA |
| *P. falciparum* | Fal-Probe | **FAM**-ACACAATGAACTCAATCATGACTACCCGTC-**BHQ1** |
| *P.vivax* | Vix-F | ACGCTTCTAGCTTAATCCACATAACT |
| *P.vivax* | Viv-R | TAATTTACTCAAAGTAACAAGGACTTCCAA |
| *P.vivax* | Vix-Probe | **HEX**-ACTTCGTATCGACTTTGTGCGCATTTTGCT-**BHQ1** |

Table S3. Results of simple logistic regression with odds ratio for being a case.

| Theme | Variable | OR |  | ROC |  | p value |  |
| --- | --- | --- | --- | --- | --- | --- | --- |
| Occupation | Farmer | 0.666 | (0.5330-0.8317) | 0.550 | (0.5189-0.5819) | **0.0019** |  |
|  | Forest worker | 5.096 | (3.404-7.894) | 0.674 | (0.6341-0.7135) | **<0.0001** | ** |
|  | Student | 0.677 | (0.4675-0.9720) | 0.548 | (0.4971-0.5996) | 0.0679 |  |
|  | Not working | 1.127 | (0.5705-2.247) | 0.515 | (0.4172-0.6127) | 0.7645 |  |
|  | Military | 3.535 | (1.263-12.51) | 0.640 | (0.5226-0.7577) | **0.0404** |  |
|  | Other | 0.189 | (0.07666-0.4009) | 0.672 | (0.6012-0.7434) | **0.0001** | ** |
| Reasons for visiting forest | Exploitation* | 1.954 | (1.586-2.413) | 0.582 | (0.5534-0.6112) | **<0.0001** | ** |
|  | Farming | 2.221 | (1.625-3.066) | 0.596 | (0.5547-0.6372) | **<0.0001** | ** |
|  | Patrol | 2.285 | (1.572-3.375) | 0.599 | (0.5500-0.6475) | **0.0002** | ** |
|  | Fishing | 2.137 | (1.362-3.431) | 0.591 | (0.5316-0.6507) | **0.0042** |  |
|  | Picked up in forest | 1.710 | (1.042-2.863) | 0.566 | (0.4970-0.6343) | 0.0674 |  |
|  | Business | 1.723 | (0.6902-4.646) | 0.566 | (0.4394-0.6935) | 0.3183 |  |
|  | Hunting | 2.417 | (0.8929-7.619) | 0.604 | (0.4750-0.7327) | 0.1399 |  |
|  | Other | 1.000 | (0.4826-2.072) | 0.500 | (0.3959-0.6041) | 1 |  |
|  | Unknown | 2.002 | (0.1915-43.09) | 0.584 | (0.2704-0.8965) | 0.6169 |  |
| Travel | To different province | 1.838 | (1.263-2.708) | 0.574 | (0.5228-0.6256) | **0.0060** |  |
|  | To different district | 2.276 | (1.635-3.207) | 0.599 | (0.5552-0.6419) | **<0.0001** | ** |
|  | To different commune | 1.980 | (1.601-2.456) | 0.584 | (0.5545-0.6132) | **<0.0001** | ** |
|  | International | 4.685 | (1.922-13.99) | 0.663 | (0.5733-0.7527) | **0.0030** |  |
|  | Non-work | 2.275 | (1.848-2.807) | 0.600 | (0.5721-0.6285) | **<0.0001** | ** |
|  | Work only | 1.838 | (1.452-2.335) | 0.575 | (0.5421-0.6075) | **<0.0001** | ** |
|  | Number of nights away* | 1.013 | (1.008-1.019) | 0.665 | (0.6404-0.6885) | **<0.0001** | ** |
|  | Visit forest | 3.462 | (2.882-4.165) | 0.650 | (0.6262-0.6745) | **<0.0001** | ** |
|  | Live in forest | 1.777 | (1.029-3.153) | 0.570 | (0.4949-0.6453) | 0.0758 |  |
|  | Days in the forest* | 1.030 | (1.024-1.037) | 0.659 | (0.6351-0.6833) | **<0.0001** | ** |
|  | Nights in the forest* | 1.042 | (1.033-1.052) | 0.669 | (0.6455-0.6933) | **<0.0001** | ** |
| Malaria history | How long been unwell (days)* | 2.577 | 2.357 to 2.828 | 0.806 | 0.7876 to 0.8249 | **<0.0001** | ** |
|  | Had recorded fever | 3.383 | 2.422 to 4.808 | 0.64 | 0.6005 to 0.6787 | **<0.0001** | ** |
|  | Times had malaria in past 1 year* | 3.292 | 2.628 to 4.168 | 0.594 | 0.5692 to 0.6189 | **<0.0001** | ** |
|  | Times people in your house had malaria in past year* | 1.269 | 1.096 to 1.477 | 0.53 | 0.5046 to 0.5551 | **0.0207** |  |
| Treatment | Had prior treatment | 4.869 | 3.847 to 6.206 | 0.68 | 0.6536 to 0.7069 | **<0.0001** | ** |
|  | Finished treatment | Model did not converge | |  |  |  | ** |
|  | Vomited treatment | 0.4971 | (0.2741-0.9247) | 0.569 | (0.4845-0.6531) | 0.0973 |  |
| Prior healthcare | Government health centre | 3.108 | (2.126-4.646) | 0.631 | (0.5854-0.6758 | **<0.0001** | ** |
| for this illness | Non-government health centre | 2.164 | (1.776-2.643) | 0.595 | (0.5675-0.6219 | **<0.0001** | ** |
|  | Government hospital | 5.04 | (1.324-32.84) | 0.668 | (0.5337-0.8016 | **0.0449** |  |
|  | Pharmacy | 1.992 | (1.619-2.458) | 0.585 | (0.5560-0.6134 | **<0.0001** | ** |
|  | Private clinic | 4.334 | (2.426-8.336) | 0.658 | (0.5984-0.7182 | **<0.0001** | ** |
| LLIN | Household has LLIN | 1.049 | (0.6827-1.614) | 0.506 | (0.4441-0.5679 | 0.8498 |  |
|  | Per person* | 0.3239 | (0.2215-0.4657) | 0.556 | (0.5311-0.5814 | **<0.0001** | ** |
|  | Use | 0.3298 | (0.2465-0.4372) | 0.632 | (0.5964-0.6673 | **<0.0001** | ** |
|  | Age (years)* | 0.9774 | (0.9666-0.9880) | 0.584 | (0.5563-0.6115 | **<0.0001** | ** |
|  | Last treated (years)* | 0.9949 | (0.9864-1.003) | 0.599 | (0.5719-0.6267 | **<0.0001** | ** |
|  | Has holes | 2.306 | (0.8517-6.809) | 0.603 | (0.4648-0.7407 | 0.157 |  |
| House | Size* | 0.9382 | (0.8859-0.9887) | 0.537 | (0.5117-0.5622 | **0.0042** |  |
|  | Sealed to mosquitoes | 1.243 | (0.4443-3.557) | 0.527 | (0.3807-0.6735 | 0.7171 |  |
|  | Have AC | 0.5427 | (0.1863-1.432) | 0.574 | (0.4408-0.7075 | 0.2917 |  |
| Personal | Use coil | 0.8569 | (0.6750-1.087) | 0.519 | (0.4850-0.5535 | 0.2704 |  |
| protection | Use repellant | 0.3994 | (0.3262-0.4879) | 0.612 | (0.5848-0.6386 | **<0.0001** | ** |
|  | Use plug-in | 0.299 | (0.2434-0.3661) | 0.644 | (0.6184-0.6704 | **<0.0001** | ** |
|  | Use LLIN | 0.3298 | (0.2465-0.4372) | 0.632 | (0.5964-0.6673 | **<0.0001** | ** |
|  | *=continuous variable | **=significant p value adjusted for multiple comparisons | | | | |  |


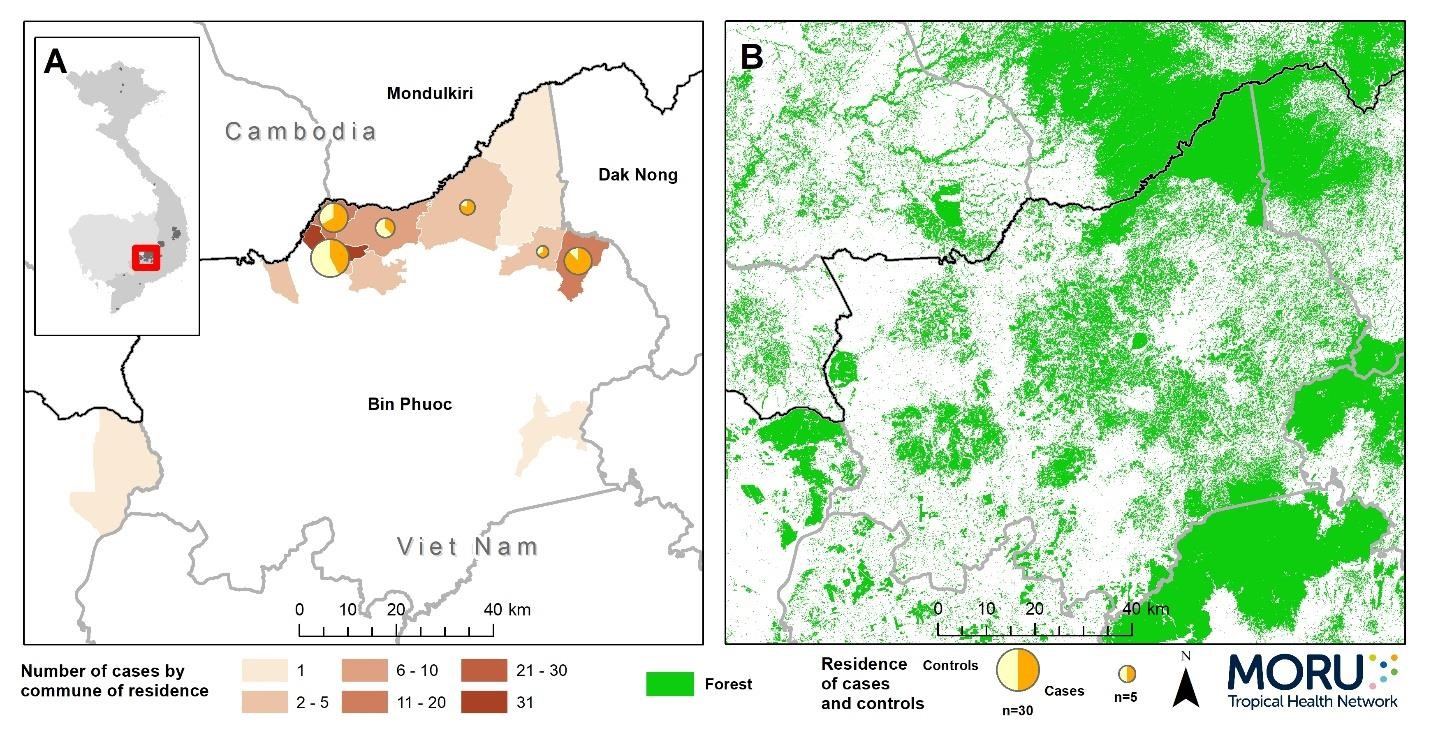


Figure S1. Commune of residence of cases and controls who visited Cambodia (A) and forest distribution (B).
